# Illegal recreational fishing causes a decline in a fishery targeted species (Snapper: *Chrysophrys auratus*) within a remote no-take marine protected area

**DOI:** 10.1371/journal.pone.0209926

**Published:** 2019-01-08

**Authors:** David Harasti, Tom R. Davis, Alan Jordan, Luke Erskine, Natalie Moltschaniwskyj

**Affiliations:** Port Stephens Fisheries Institute, NSW Department of Primary Industries, Nelson Bay, NSW, Australia; University of Auckland, NEW ZEALAND

## Abstract

One role of Marine Protected Areas is to protect biodiversity; however, illegal fishing activity can reduce the effectiveness of protection. Quantifying illegal fishing effort within no-take MPAs is difficult and the impacts of illegal fishing on biodiversity are poorly understood. To provide an assessment of illegal fishing activity, a surveillance camera was deployed at the Seal Rocks no-take area within the Port Stephens-Great Lakes Marine Park from April 2017-March 2018. To assess impacts of illegal fishing activity in the no-take area, Baited Remote Underwater Video Systems (BRUVs) were used to quantify abundance and size of snapper *Chrysophrys auratus* from 2011–2017. BRUVs were also deployed at two nearby fished locations and two other no-take areas to allow comparison. Over 12 months of camera surveillance, a total of 108 recreational vessels were observed illegally fishing within the no-take area (avg 9.0 ± 0.9 per month). The greatest number of vessels detected in a single month was 14 and the longest a vessel was observed fishing was ~ 6 hours. From 2011–2017, the abundance of *C*. *auratus* within the Seal Rocks no-take area significantly declined by 55%, whilst the abundance within the other fished areas and no-take areas did not significantly decline over the same period. Lengths of *C*. *auratus* in the Seal Rocks no-take area were significantly smaller in 2017 compared to 2013 which was driven by a decline in the number of legal sized fish over 30 cm. Based on mean number of illegal fishers per vessel recorded in the no-take area, and an allowable bag limit of 10 *C*. *auratus* per person, it is possible that more than 2,000 *C*. *auratus* are removed annually from this no-take area. There is a strong likelihood that illegal recreational fishing is causing a reduction on a fishery targeted species within a no-take MPA and measures need to be implemented to reduce the ongoing illegal fishing pressure.

## Introduction

Marine Protected Areas (MPAs) provide conservation benefits to many of the species and habitats within their boundaries [1, 2]. However, not all MPAs meet their conservation objectives [3] as the response of species to protection within MPAs is influenced by a range of factors, including age and size of the MPA [4, 5], degree of isolation [6], extent and types of habitats [7, 8], and enforcement of regulations about activities that are illegal within the boundaries [9, 10].

No-take areas are of particular significance in MPAs as they generally provide the highest level of protection due to the removal of fishing pressure, and are often identified as providing valuable reference areas to evaluate changes in biodiversity when harvest is removed [11]. However, their value is affected by adherence of the rules by fishers and by formal enforcement of regulations. Unfortunately, voluntary adherence to the rules by fishers of MPA rules combined with minimal formal enforcement often results in illegal activities, such as fishing, contributing to reduced effectiveness in meeting their conservation objectives [12]. Fishing within no-take areas is a problem world-wide, with numerous studies demonstrating various levels of illegal fishing often occurs within these areas [13, 14]. Declines in species abundance, particularly that of target species, and diversity occurs in no-take areas with declines attributed to illegal fishing activities [12, 15]. The direct impacts of illegal fishing on fishery species in no-take areas are seldom quantifiable, however, poaching was shown to cause a decrease in species such as abalone [16], limpets [15], and target fish species [13, 17], although the magnitude of change is strongly influenced by the life history characteristics of the exploited species [18]. MPAs that are considered to have good enforcement are known to have a greater positive influence on target fish abundance, particularly within no-take areas compared to fished areas [19], whilst areas with weak enforcement are less likely to achieve their conservation objectives [6,9].

While many factors that contribute to the success of an MPA have been well documented [10, 20]; very little effort has been directed to quantifying the potential impacts of levels of illegal fishing within MPAs, which can both reduce the effectiveness of an MPA in achieving the key objectives of conservation [21], and use as scientific reference sites. Non-compliant fishing activity in no-take areas is generally difficult to assess and quantify as vessels generally actively try to avoid detection. Assessment methods for quantifying illegal fishing include aerial surveys [17, 22], estimates of discarded fishing gear [23, 24], and fisher interviews [25, 26]. More recently, with advancements in camera technology, remotely deployed cameras are being used to quantify fishing activity around features such as artificial reefs [27, 28], and to assess illegal fishing activity and effort [29–31].

The aim of this study was to evaluate whether illegal fishing activity within a no-take area can significantly reduce the relative abundance and size of pink snapper (*Chrysophrys auratus*), the most important recreational fishery targeted species in the Port Stephens-Great Lakes Marine Park (PSGLMP); a large multi-use marine park in temperate waters on the east coast of Australia. *Chrysophrys auratus* are a dominant species on shallow reefs, and are important predators in structuring shallow reef communities [32]. Apprehension of vessels found illegally fishing within no-take areas in PSGLMP has identified that *C*. *auratus* is the most commonly targeted species by illegal fishing activities (NSW DPI unpublished data). *Chrysophrys auratus* responds positively within MPAs, with evidence of increase in abundance and size within no-take areas [33, 34]. To assess the influence of illegal fishing on the abundance and length of *C*. *auratus* within a no-take area, remote camera surveillance data were used to quantify the amount of illegal fishing activity occurring within a no-take area, and determine variables that influence illegal fishing activity, whilst Baited Remote Underwater Video systems (BRUVs) were used to assess changes in abundance and size of *C*. *auratus*. This study provides evidence on the need to reduce illegal fishing in areas that are established to remove the stressors of harvest, bycatch, and incidental mortality.

## Materials and methods

### Ethics statement

This study was conducted in accordance with the Port Stephens-Great Lakes Marine Park requirements for non-extractive research within no-take areas. Research was conducted under NSW Department of Primary Industries Scientific Permit: P01/0059(A)-2.0. This research did not involve any endangered or protected species and no animals were sampled. This study was conducted in compliance with the NSW DPI Animal Care and Ethics Committee permit: 10–09 (Monitoring of fish communities using visual and video surveys). The installation and operation of the surveillance camera was approved by the Australian Maritime Safety Authority via a property access request in January 2017.

### Study area

The focus of this study is the Seal Rocks no-take area within the Port Stephens-Great Lakes Marine Park in New South Wales, Australia (32°27'39.36"S 152°33'34.94"E). The PSGLMP was declared in 2005 with the marine park zoning plan implemented in 2007. This no-take area is the largest within the marine park at 6,580 hectares, equating to 37% of no-take area across this MPA. The Seal Rocks no-take area is the most remote no-take area within the MPA as there are no major boat ramps or towns nearby. This no-take area is the most important protected area within the marine park as it contains high levels of biodiversity and a number of threatened species i.e black rock cod [35]. As a result, it is an important scientific reference location for monitoring the marine estate [36, 37]. The Seal Rocks no-take area contains some of the highest recorded relative abundances of *C*. *auratus* within Australia using baited video methods [37]. Whilst fishing pressure should decline to zero following the establishment of a no-take area, increased amounts of fishing tackle on the seafloor within the no-take area observed during research surveys in 2012–2013 indicated that illegal fishing was occurring and research surveys to the location often observed vessels illegally fishing (author’s observations).

### Surveillance camera

**Fig 1:**
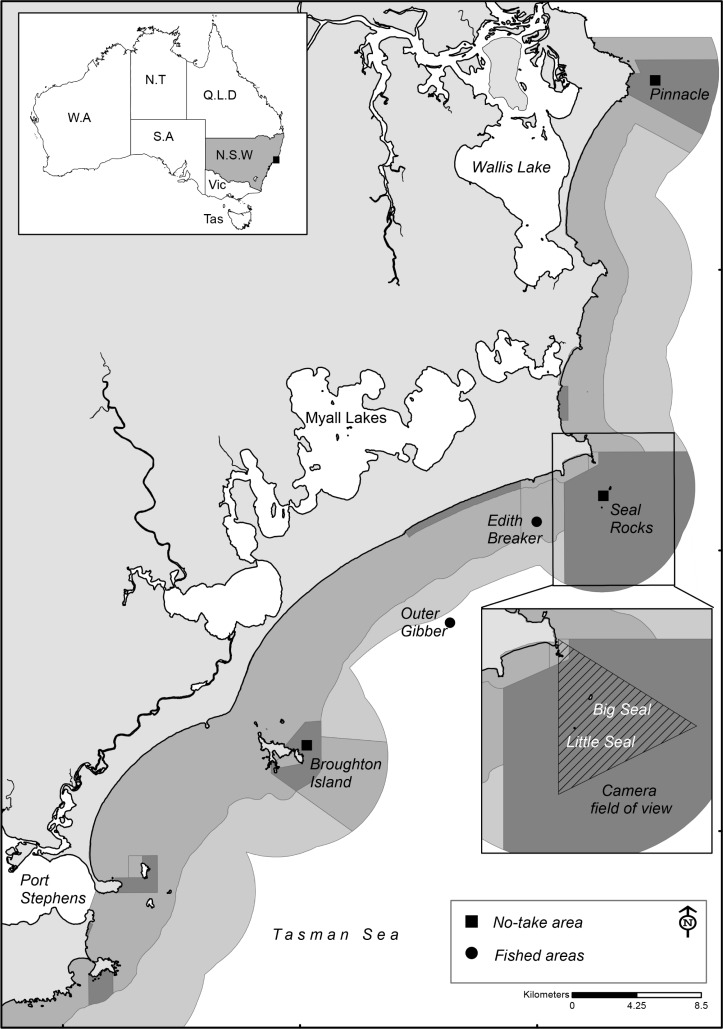
Location and field of view of the surveillance camera at Seal Rocks (crossed area is area of camera coverage) and locations for Baited Remote Underwater Video surveys of snapper (*Chrysophrys auratus*). Dark grey areas = no-take areas, mid grey = partially protected areas and light grey = General use areas.

In March 2017, a Canon VB-M50B surveillance camera was installed in a CoastalCOMS housing (https://www.coastalcoms.com) on the Seal Rocks lighthouse at Sugarloaf Point. The camera was positioned to provide video footage of the Seal Rocks no-take area, particularly the two rocky outcrops (Big Seal and Little Seal), allowing the recording of all vessel activities within this part of the no-take area. This area contains reef features that are often targeted by fishers (Fig 1). The distance from the camera to Big Seal Rock is ~ 2.7 km and ~ 3.8 km to Little Seal Rock. The camera was programmed to conduct a ‘tour’ of the Seal Rocks no-take area, which involved the camera panning and zooming from the north to the south section of the no-take area. Each camera tour took approximately 2 mins and was repeated continuously throughout the day between 0530 to 2100. Each day the footage was stored and maintained on a cloud accessible hard drive for 60 days. Camera footage was accessed using Milestone XProtect Smart Client software to allow playback of recorded footage.

The camera footage was analysed from 1 April 2017 to 31 March 2018 (365 days) to provide an assessment of the presence of vessels within the no-take area, with data aggregated on a calendar monthly. Footage was retrieved at 30 min increments (i.e. 6:00 am, 6:30 am, 7:00 am), with a 2 minute camera tour watched for the presence of any vessels at each time increment. If a vessel was detected, extended analysis was undertaken of the footage to determine the time and direction of the vessels arrival and departure from the no-take area. Analysis of the camera data commenced as soon as there was enough sunlight to observe vessels and ceased when it was too dark to detect vessels. While night time fishing may be occurring, it was not possible to detect with this camera system.

Sea state was recorded daily as calm, moderate, rough, and very rough based on wave action over the two rocky outcrops (Big and Little Seal). The position where the vessel was observed in the no-take area was recorded and the approach/departure direction of vessels entering the no-take area was recorded as from the north or the south.

While it was not always possible to directly observe fishers engaging in illegal fishing activity for vessels in the Seal Rocks no-take area (due to the resolution of the camera system and distance of some of the vessels from the camera), fishing activity was assumed for vessels that were stationary for > 10 m in the no-take area and which were not conducting permitted activities such as sailing, scuba diving, fisheries compliance, or research. Ten minutes was deemed long enough for a fisher to commence fishing as when vessels were close enough to the camera for detailed observation it was observed that fishing mostly commenced in < 5 mins. The time that a boat first arrived to the time of its departure was recorded as fishing effort in hours-minutes. For those vessels that were close enough for details to be discerned, a determination was also made if the illegal vessel was considered to be a recreational or commercial fisher based on the vessel appearance, registration and the activity being undertaken.

To deter the ongoing illegal fishing activity within the no-take area, a media campaign was conducted between 27–30 September 2017 (about the mid-point of the study) to alert the public to the presence of surveillance cameras within the marine park. Articles were published in local newspapers on the presence of the camera, including details of recent infringement notices issued to fishers for illegally fishing in the Seal Rocks no-take area.

### Baited Remote Underwater Video

Baited Remote Underwater Video (BRUV) surveys were conducted in the Seal Rocks no-take area, other no-take areas in the PSGLMP, and nearby fished areas (Fig 1) between 2011 and 2017 (excluding 2012, 2014) as part of a larger research program assessing fish assemblages on rocky reefs, with sampling conducted July—August for each year surveyed. Sampling within the Seal Rocks no-take area involved four BRUVs deployed around each of Big Seal rock and Little Seal rock (Fig 1 insert). The placement of the BRUVs was haphazard, with BRUVs deployed approximately 200 m apart and on rocky reef habitats in depths of 20–50 m. The other two no-take areas sampled in the PSGLMP were the Pinnacle (Forster) and Broughton Island, as these sites are the no-take zones located to the immediate north and south of Seal Rocks, with eight replicate BRUV drops for each no-take area for each sampling occasion. The Forster Pinnacle location is approximately 26 km from Seal Rocks whilst Broughton Island is 24 km. To compare relative abundance of *C*. *auratus* at sites open to commercial and recreational fishing, the two closest reef systems, Edith Breakers reef (~3 km from Seal Rocks) and Outer Gibber reef (~11 km from Seal Rocks) that are of similar depth to Seal Rocks, were sampled at the same time periods also using BRUVS with eight haphazardly selected deployments for each location (Fig 1).

BRUVS were deployed for 30 mins as per the methods described in [38]. From 2013 onwards, stereo-BRUV units replaced the single BRUV units enabling *C*. *auratus* lengths to be measured (total length). BRUV units consisted of Canon HG21 and HG25 video cameras with wide angle lens that were housed in custom-made SeaGIS Pty Ltd. housings (http://www.seagis.com.au). Stereo BRUVs were calibrated prior to each sampling period following the methods detailed by [39]. Camera calibrations were checked regularly with scale bars of known lengths to ensure measuring accuracy. Bait consisted of approximately 1 kg of crushed pilchards (*Sardinops neopilchardus*), mashed into a plastic mesh bait bag attached to the end of each bait-pole (~1.5 m distance from the frame).

Following the 30 minute set, BRUV units were retrieved and video files were downloaded and analysed using SeaGIS Eventmeasure software (version 3.1–4.1). The MaxN of *C*. *auratus* was estimated for each 30min period, where MaxN is an indication of relative abundance of *C*. *auratus* and is estimated as the maximum number (MaxN) of *C*. *auratus* in the frame at any one time during each set [40]. To minimise the effects of water visibility on relative abundance (MaxN) of *C*. *auratus*, the field of view was standardised to approximately 3 m behind the bait bag estimated visually by the video analyser for the single BRUV units and using stereo distance measurements in Eventmeasure for the stereo-BRUVs. The length of each *C*. *auratus* observed at the time of MaxN was measured as total length (tip of fish nose to tip of tail fin) and the minimum size limit for *C*. *auratus* in NSW is 30 cm (total length).

### Statistical analysis

Temporal variability in the number of vessels seen illegally fishing was analysed using a two-way ANOVA with time of day and time of week as explanatory variables. Time of day was split into 2 hour periods from 05:00 to 21:00 and time of week was Monday-Sunday. If the ANOVA was significant, then SNK post-hoc test was used to determine where the differences among occurred. To assess if the media campaign (27–30 September 2017) influenced the presence of illegal fishing vessels, a one way ANOVA analysed number of vessels in three-month periods (April 2017—June 2017, July 2017—Sept 2017, October 2017—December 2017 and January 2018—March 2018) with a SNK test determining where the differences occurred.

### Relative abundance of *C*. *auratus*

To assess changes through time in the mean relative abundance of *C*. *auratus* at each of the five locations, a one-way ANOVA was used. Where there was a significant difference through time, a linear regression analysis was used to determine the rate of change. As in each year there are multiple BRUV deployments (n = 40), the regression analysis needed to be adjusted for variation within each year, therefore a regression for multiple y-values was used with r^2^ indicated [41].

### Comparison of lengths of *C*. *auratus*

Total lengths for *C*. *auratus* were aggregated within 2 cm bins by year (2013 and 2017) to generate a length frequency distribution. To assess differences in length frequency for *C*. *auratus* (total length) between 2013 and 2017 within the Seal Rocks no-take area, a Kolmogorov-Smirnov test was conducted. This test calculates the maximum distance between two cumulative distributions (test statistic D), and determines a corresponding P value.

To assess differences between years (2013, 2015, 2016 and 2017) in mean size of MaxN recorded *C*. *auratus*, a single factor ANOVA was conducted with year as a fixed factor. The same ANOVA analysis was used to assess differences in mean size of *C*. *auratus* > 30 cm and mean size of the largest observed *C*. *auratus* per stereo-BRUV deployment (as described in [37]). Post-hoc SNK comparisons were conducted to assess differences in *C*. *auratus* lengths between years for each location.

### Compliance effort

To quantify the potential impact of how differing levels of formal compliance effort could influence the abundance *of C*. *auratus* in the Seal Rocks no-take area, analysis was undertaken between two different time periods where formal compliance effort was considered high (2010 and 2011), compared with moderate effort (2015 and 2016). The level of compliance effort was based on the number of offshore patrol days for these years recorded from compliance vessel logbooks. Baseline compliance effort was determined for 2015 and 2016, whilst 2010 and 2011 were considered to be periods of greater compliance effort with more targeted compliance in the early stages of marine park implementation. Greater compliance effort was described as double the number of offshore patrol days compared to ‘baseline compliance’. Analysis was based on a single fixed factor (year) analysis of variance comparing *C*. *auratus* relative abundance between 2010 and 2011 combined with 2015 and 2016 combined.

All statistical analysis was conducted in IBM SPSS Statistics version 25 (www.ibm.com/spss).

## Results

### Camera surveillance

**Fig 2:**
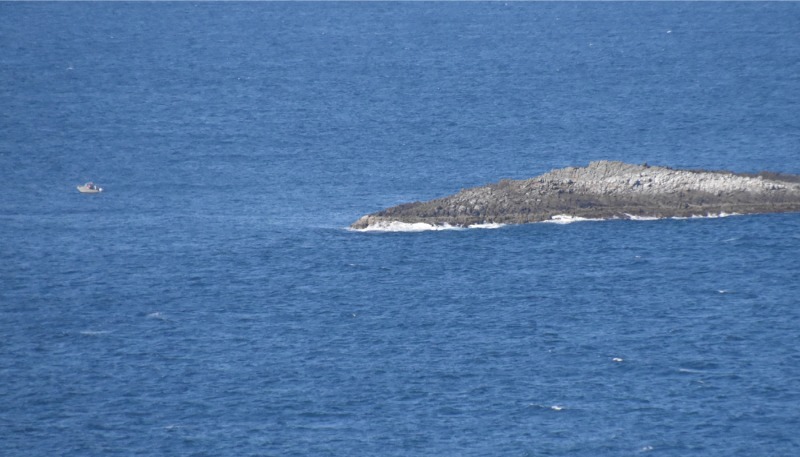
Vessel illegally fishing within the no-take area at the northern end of Big Seal Rock. When the camera was zoomed in, three people were observed illegally fishing from this vessel.

A total of 108 vessels were detected illegally fishing within the no-take area between 1 April 2017 and 31 March 2018 (see Fig 2 for example of vessel illegally fishing). All the vessels were determined to be recreational fishing vessels with no vessels identified as commercial vessels. Vessels were present on 47% of calm days; 17% of moderate days; and 3% of rough days, with no vessels detected when conditions were classified as very rough. Of the 108 illegal fishing vessels, 46% approached the no-take area from the south, 48% from the north, and approach direction unclear for the remaining 6% of vessels.

**Fig 3:**
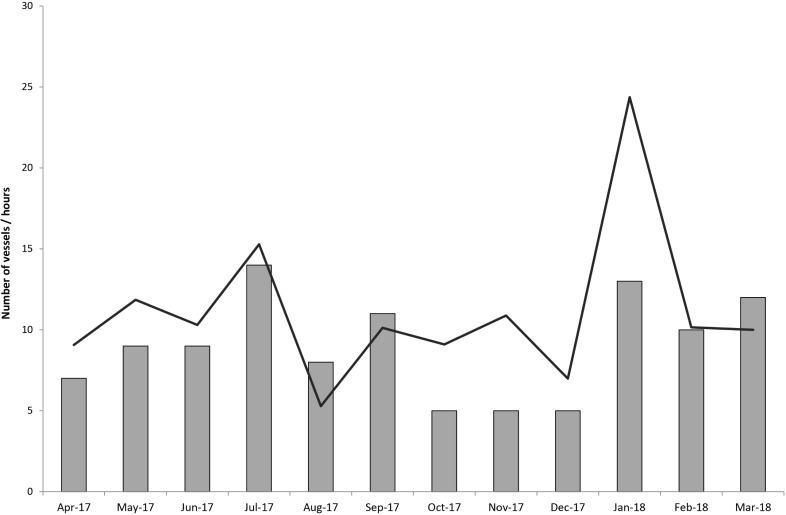
Number of vessels observed illegally fishing from April 2017 to March 2018 within the Seal Rocks no-take area (grey bars) for each month, and the number of hours observed fishing effort (dark line).

The mean number of vessels detected illegally fishing over the 12 months was 9.0 ± 0.9 S.E per month. The greatest number of vessels recorded fishing in a single month was 14 vessels in July 2017 (Fig 3). The fewest number of vessels seen fishing was in October, November, and December 2017 with only 5 vessels each month; this was the 3-month period following the public education campaign regarding the camera presence at Seal Rocks. Pre announcement of the presence of the camera (April 2017 –September 2017), the mean number of vessels illegally fishing from April–June 2017 was 8.3 ± 0.6 and July–September 2017 was 11 ± 1.7 compared with post camera announcement with mean 5 ± 0 vessels in October–December 2017 and 11.6 ± 2.3 for January–March 2018. The difference in illegal fishing activity between the 3 month periods was significant (F_3,11_ = 8.70, P < 0.006), with post-hoc test indicating that the three months following the surveillance camera media announcement (October–December 2017) having significantly fewer illegal fishing vessels than the other three periods.

From 1 April 2017–31 March 2018, a total of 133 hours of fishing effort was recorded within the no-take area (Fig 3). The month that had the highest recorded fishing effort was January 2018 with approximately 24 hours fishing effort observed. The mean number of hours spent illegally fishing within the no-take area over the 12 months was 11.0 ± 1.4 hours per month. The longest period a vessel was recorded fishing in the no-take area occurred on Friday 24 November 2017 when a vessel was observed from 6:32 am to 12:26 pm; a total time of 5 hr and 54 mins. The average time a vessel spent illegally fishing within the no-take area was 1:14 min ± 6 mins.

**Fig 4:**
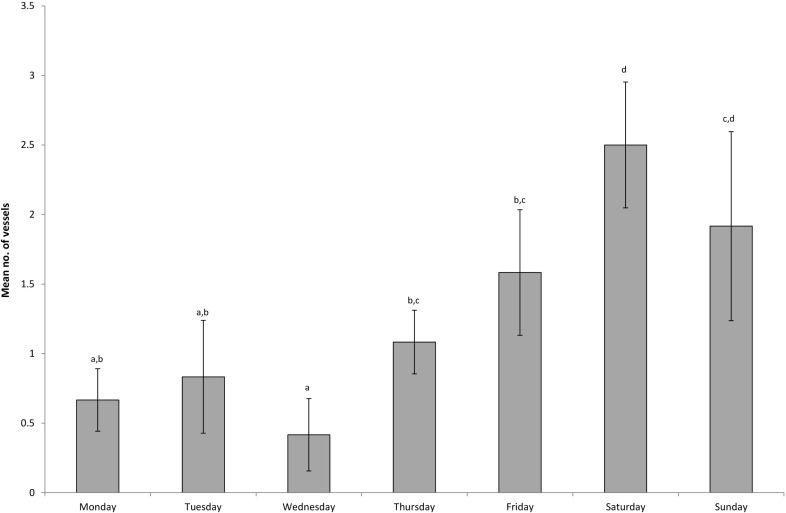
Mean number of vessels (+/- standard error) detected within Seal Rocks no-take area for each day of the week. Means with the same letter are not significantly different from one another.

**Fig 5:**
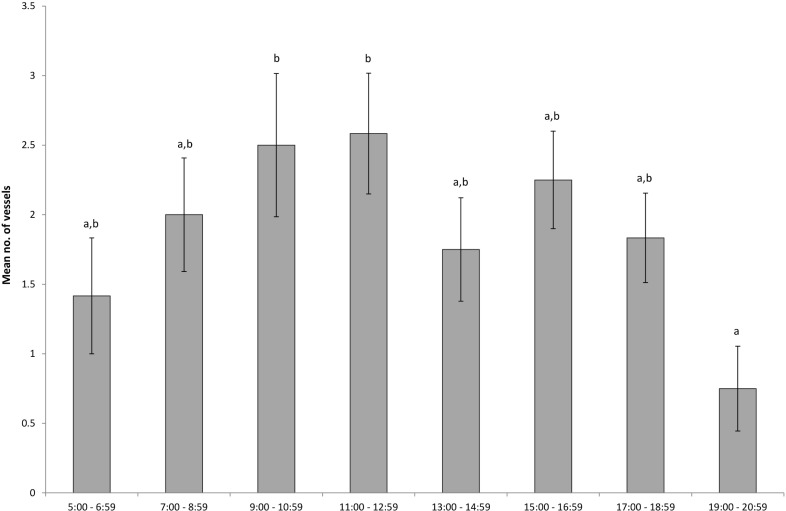
Mean number of vessels (+/- standard error) detected within Seal Rocks no-take area for time of day periods per month. Means with the same letter are not significantly different from one another.

The mean number of vessels illegally fishing differed significantly among days of the week (F_6,672_ = 7.96; P < 0.001), and differed by time of day (F_7,672_ = 2.11; P < 0.041); however, there was no interaction between the two factors (F_42,672_ = 0.83; P > 0.774). The average number of vessels detected was greatest on Saturdays compared with weekdays, but no different from Sundays (Fig 4). While the average number of vessels detected on Wednesdays was less than Fridays, Saturdays and Sundays, it was not different from other weekdays. The time of day that vessels were detected was very similar from 05:00 through to 18:59, however the average number of vessels present in the no-take area from 09:00–12:59 was more than three times greater than after 7pm (Fig 5).

### Illegal take of *Chrysophrys auratus*

Records of all compliance interactions of vessels illegally fishing within the Seal Rocks no-take area from 2010–2017 recorded a mean of 2.14 ± 0.16 persons per vessel. Based on the mean of 9.0 vessels per month recorded from the surveillance camera, using an average of 2.14 person per vessel, if each fisher took the bag limit of 10 *C*. *auratus* each occasion, it is estimated that the number of vessels illegally fishing could have caught up to 192 *C*. *auratus* (9 vessels x 2.14 persons x 10 *C*. *auratus* per person) in the no-take area each month; this equates to the potential removal of 2,304 *C*. *auratus* over 12 months.

As an example, on three vessels intercepted by fisheries compliance during the 12 month camera surveillance period, 79 illegally caught fish were seized (with 3, 2, 2 fishers on each vessel). The majority of the illegal catch as recorded by compliance officers using common names was pink snapper (65%), and also included other species such as tailor (20%), flathead (>1%%), pearl perch (>1%%), bonito (>1%%), goatfish (>1%%) and samson fish (>1%). Of the seized *C*. *auratus*, all were greater than the legal size limit of 30 cm and ranged in size from min 30 cm to max 75.3 cm.

### Differences in relative abundance and lengths of *C*. *auratus*

**Fig 6:**
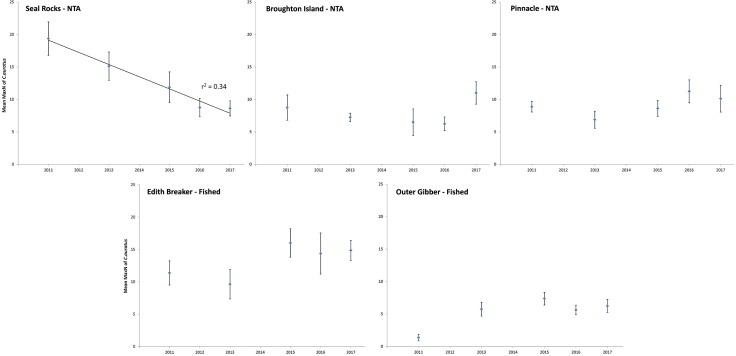
Changes through time (years) in mean relative abundance of *C*. *auratus* (+/- standard error) for each location. Goodness-of-fit measure for significant linear regression models indicated by r^2^ value.

A total of 200 BRUV deployments between 2011 and 2017 detected 1,945 individual *C*. *auratus* across the five locations and five sampling years. The average relative abundance of *C*. *auratus* changed through time at some locations, but the nature of the change depended on the location (Fig 6). The Seal Rocks no-take area was the only location where average abundance of *C*. *auratus* significantly differed among years (F_4,39_ = 5.12; P = 0.002); there was evidence that there was a linear decline (F_1,3_ = 41.55; P = 0.008) with a 55% decline in MaxN abundance from 2011 to 2017 at a rate of 2.8 fish lost per year since 2011. Of the fished locations, only Outer Gibber had a significant change in the average abundance of *C*. *auratus* among years (F_4,39_ = 6.76; P<0.001), while the abundance increased by 84%, there was no evidence that this was a linear change through time. None of the other locations (fished or unfished) had a significant change in average abundance of *C*. *auratus* among the five years (Pinnacle at Forster: F_4,39_ = 1.20; P = 0.324, Broughton Island: F_4,39_ = 1.55; P = 0.208, Edith Breaker: F_4,39_ = 1.35; P = 0.272).

**Fig 7:**
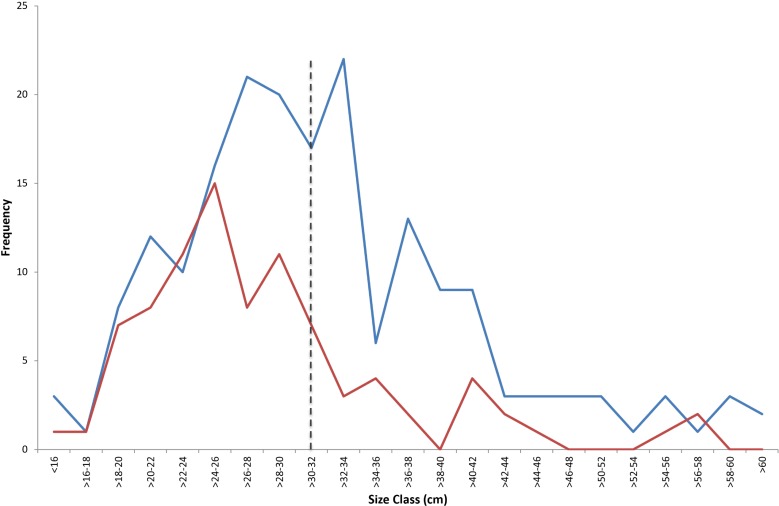
Comparison of length frequency distribution (total length) for MaxN recorded *C*. *auratus* in 2 cm bins in the Seal Rocks no-take area between 2013 (blue line) and 2017 (red line). Dashed black line = minimum legal size for *C*. *auratus* (>30 cm).

**Fig 8:**
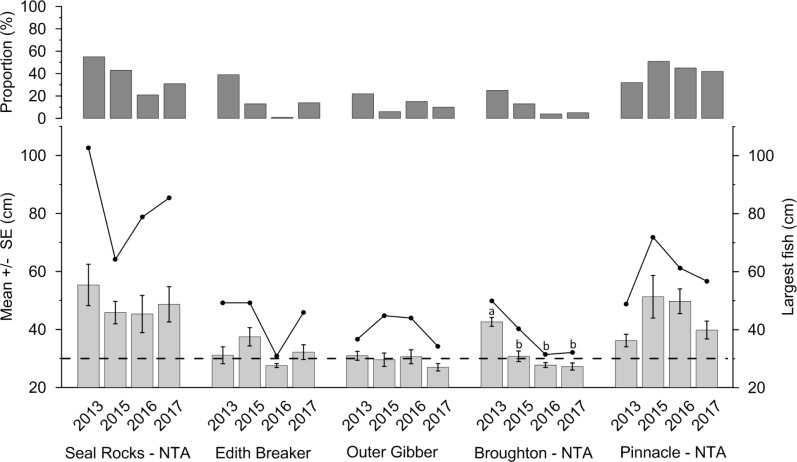
Mean total length (± S.E.) of largest observed *C*. *auratus* per BRUVs deployment for each location across four years (light grey bars). Largest *C*. *auratus* (total length–cm) observed for each location for each of the four years sampled (black lines). Years with the same letter are not significantly different from one another within each location (SNK analysis). No letters indicate years are not significantly different within each location. Proportion of *C*. *auratus* over the legal size limit (> 30 cm) indicated by dark grey bars. Legal size limit (30 cm) indicated by dashed black line. NTA = No-take Area.

As Seal Rocks was the only location to show a significant decline in abundance through time, changes in size frequency distribution between the first measurements recorded with stereo-BRUVS (2013) and the last survey (2017) were examined. There was a significant difference in the length frequency distributions between 2013 (n = 187) and 2017 (n = 88) (D statistic = 0.2293; P<0.005, Fig 7). In 2013, the dominant size class for *C*. *auratus* was 32–34 cm, compared to a size class of 24–26 cm in 2017. Numbers of individuals were similar for fish under < 26 cm for 2013 and 2017, whilst there was a decline in the number of individuals > 26 cm within the no-take area in 2017. For the Seal Rocks no-take area, the proportion of *C*. *auratus* over legal size in 2013 was 55% compared with only 21% in 2016 and 31% in 2017 (Fig 8). Similar declines in proportion of legal sized fish was also observed for Edith Breaker, Outer Gibber and Broughton Island, whilst the Pinnacle no-take area had a higher proportion of legal sized *C*. *auratus* in 2015, 2016 and 2017 compared with 2013.

Based on the largest *C*. *auratus* observed per stereo-BRUV drop, Seal Rocks had the largest observed fish in 2013, 2016 and 2017 whilst the Pinnacle had the largest fish in 2015 (Fig 8). The Pinnacle had significantly larger fish in 2015 and 2016 compared with 2013, whilst 2013 and 2017 were similar. There was a significant difference in the largest sized *C*. *auratus* for Broughton Island with the largest *C*. *auratus* observed being significantly smaller in 2015, 2016 and 2017 compared with 2013 (Fig 8).

**Fig 9:**
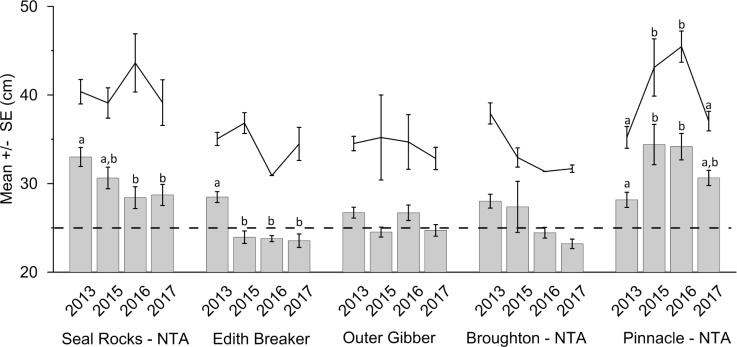
Mean total length (± S.E.) of all observed *C*. *auratus* per BRUVs deployment for each location across four years (grey bars). Mean total length (± S.E.) of *C*. *auratus* > 30 cm observed for each location for each of the four years sampled (black lines). Years with the same letter are not significantly different from one another within each location (SNK analysis). No letters indicate years are not significantly different within each location. Legal size limit (30 cm) indicated by dashed black line. NTA = No-take Area.

Within the Seal Rocks no-take area, the mean size of all *C*. *auratus* was found to decrease from 2013 to 2017. Fish in both 2016 and 2017 were significantly smaller than 2013, whilst 2013 and 2015 were similar (Fig 9). Both Edith Breaker and Outer Gibber had significant differences in mean size of all *C*. *auratus* between years, with *C*. *auratus* at Edith Breaker being significantly smaller in 2015, 2016 and 2017 compared with 2013. The Pinnacle no-take area was the only location to see an increase in the mean size of both all sized *C*. *auratus* and *C*. *auratus* > 30 cm with the largest sizes recorded for both classes in 2015 and 2016 (Fig 9).

### Compliance effort

There was a significant difference in the relative abundance *of C*. *auratus* at Seal Rocks between periods of greater compliance effort (2010 and 2011) and baseline compliance effort (2015 and 2016) (single-factor ANOVA: F_1,31_ = 12.81, P < 0.001). Relative abundance of *C*. *auratus* in 2015 and 2016 combined (mean: 10.3 ± 1.4) declined by 43% when compared with 2010 and 2011 combined (mean: 18.0 ± 1.6).

## Discussion

This study documented a decline in *C*. *auratus* abundance within a no-take area, despite *C*. *auratus* abundance increasing or stable at fished and other no-take areas in close proximity (~3 km to closest fished location). This suggests that the decline of *C*. *auratus* within the Seal Rocks no-take area is not driven by broader environmental variables. This decline is also in conflict with evidence that both the abundance and length of *C*. *auratus* increases within no-take areas in NSW [37, 42, 43], and in many locations across its range [44]. Additionally, it has been previously established through acoustic tracking surveys that *C*. *auratus* display strong site fidelity and have small home ranges within the PSGLMP [45]. Therefore, it is highly unlikely that fish are emigrating from the Seal Rocks no-take area as this level of emigration was not evident at other sites surveyed within this study. However, localised movements of *C*. *auratus* around Seal Rocks are yet to be quantified and warrant further investigation.

Adherence to the rules that define allowable activities within MPAs is a key component in ensuring the conservation objectives of these areas are met. This study demonstrated that a long-range live-feed surveillance camera is a useful tool to assess the occurrence of illegal fishing activities within a no-take area. The twelve months of camera operation revealed that illegal recreational fishing frequently occurred within the Seal Rocks no-take area in the Port Stephens-Great Lakes Marine Park (PSGLMP). Based on compliance infringement data from 2007–2013, a high level of illegal fishing activity occurs throughout the PSGLMP [46]; however, this research did not provide details on where illegal fishing activity was occurring. Our study indicates that despite the marine park being in place since 2007, not all recreational fishers are adhering to marine park regulations within the Seal Rocks no-take area, and are willing to risk being caught fishing illegally.

Fishers have expressed difficulty in identifying marine park boundaries [47], however, the Seal Rocks no-take area has been enforced since 2008; a period of ten years for fishers to be aware of zone boundaries. In the initial implementation of the PSGLMP zoning plan (2008–2011), offshore compliance effort was considered to be high with more dedicated compliance patrols, which also coincided with the higher abundance of *C*. *auratus* within the Seal Rocks no-take area. During the implementation period, a large number of infringement notices were issued to illegal fishers in the PSGLMP [46], with these monetary fines and regular compliance presence acting as a deterrent to illegal fishing. It is highly unlikely that the illegal fishing activity in the area could be considered accidental, given considerable public education over the past decade to ensure fishers are aware of the no-take areas within the PSGLMP. This has been supported by the production of readily available hard copy and web-based maps of zoning arrangements, boat based electronic maps, as well as signage at all boat ramps within the marine park.

Contrary to other studies that have shown that targeted fishery species increase in abundance with distance from human population centres [6], this study indicates that the remoteness of the Seal Rocks no-take area may be hindering its effectiveness. This likely reflects the fact that it is difficult for compliance vessels to approach without being observed by illegal fishing vessels that will rapidly leave the area when a compliance vessel approaches. Such behaviour was observed several times on the surveillance camera. In addition, distance from the main boat ramps of Forster and Port Stephens, with a minimum of ~90 mins required to arrive at the Seal Rocks site, makes it more of a difficult area to patrol compared to closer areas. These factors make it easier for illegal fishing at this site as there is a lower risk of detection compared to many other no-take areas in the marine park. Fishers’ primary motivations for illegal fishing are perceived higher catches in no-take areas and a low probability of detection [25]. These site factors differ from other no-take areas in the PSGLMP (i.e. Cabbage Tree Island, Broughton Island) which are less ‘open’, with compliance vessels being able to approach covertly from behind the islands and also more accessible with patrols only requiring ~ 30 mins to reach the site.

An inherent challenge that marine park managers face is the difficulty in quantifying levels of illegal fishing within no-take areas [21]. In addition to the camera being a valuable tool for estimating illegal fishing activity, it is also a useful tool for fisheries compliance. The camera system provides fisheries compliance staff with a constant live video feed of the no-take area, which can assist in operations to intercept illegal fishing vessels, which occurred on several occasions during this study. This live feed allowed fisheries compliance vessels to respond in real-time and change their approach angles to assist with illegal vessel intercepts. The ability of the camera to store recorded footage also provided fisheries compliance with evidence for post-incident investigations and assisted in interviews with illegal fishers particularly in regards to vessel movements and activity within the no-take area.

Whilst 108 vessels were detected during the 12 month surveillance period, this is considered an under-estimate of the illegal fishing activity within the no-take area as there are limitations with the camera system. The camera could not detect illegal activity at night, even though it was obvious vessels were fishing during dark given their presence in the no-take area at sunrise and their continued observed presence in the no-take area on sunset. On several occasions, vessels were observed departing the no-take area on sunrise, indicating they had potentially been fishing at the site during darkness. As the camera was only assessed on 30 min intervals, there is the small chance that vessels may have been missed if they fished for < 30 mins between monitoring intervals (i.e. they were present from 10:01–10:29). It was also difficult to see vessels in limited visibility, particularly if it was raining and during dawn and dusk. It was also very hard to detect small vessels in moderate-rough seas or if they were hidden behind Big Seal rock. With future technological advancements, these types of cameras may have increased lens magnification and other functionalities that will help reduce constraints [28].

We found that illegal fishing activities at Seal Rocks were focussed on weekends and on periods where sea conditions were calm or moderately calm, and identified that fishing was most likely to occur during the late morning and late afternoon periods. On the Great Barrier Reef between 3–18% of fishers admitted to poaching within the past year and that poaching activities occurred in specific localities and during holidays [25]. This highlights the potential for using data on patterns in illegal activity to focus compliance effort allowing maximise effectiveness of patrols and thereby reduce infringement levels, and also create a suitable deterrence effect for repeat offenders.

Most recreational fishers view illegal fishing as personally and socially unacceptable on the Great Barrier Reef [48], although illegal fishing occurs in its no-take areas [21]. Four main factors encourage poaching behaviour in recreational fishers: pluralistic ignorance, false consensus, social learning, and a perceived lack of deterrence [48]. While the majority of recreational fishers in the PSGLMP support the marine park, 45% of fishers believed that some form of illegal fishing occurred within no-take areas [49]. Addressing these issues to reduce ongoing illegally fishing activity within the Seal Rocks no-take area and fishers’ perceptions about poaching within the PSGLMP will require a range of management responses, particularly given that some recreational fishers are opposed to the marine park [50]. During this study, a widely publicised community education campaign about the presence of the Seal Rocks camera resulted in significant drop in vessel numbers for the three months following the camera announcement, however, fishers were still observed frequently illegally fishing within the no-take area. This supports the prioritisation of enforcement ahead of other marine park management changes, such as future expansion, and that strategically concentrating enforcement effort produces the greatest conservation benefits [51].

Whilst the no-take area at Broughton Island is considered to have more regular compliance patrols, due to its close proximity to major boat ramps, it is known that illegal fishing is also occurring within this location based on fisheries compliance data (Fisheries compliance *unpublished data*). The length and abundance data for *C*. *auratus* from 2011–2017, particularly the significant decline in the largest size *C*. *auratus*, indicates that there could also be ongoing illegal fishing activity with the Broughton Island no-take area as abundance and lengths of *C*. *auratus* are similar or less than nearby fished locations. An assessment of illegal fishing activity within the Broughton Island no-take area warrants further investigation and it could be of use to use surveillance cameras at this location to gain an understanding of how much illegal fishing is occurring. Of less concern is the Pinnacle no-take area as the mean size of *C*. *auratus* (all fish) and the mean size of fish > 30 cm has increased from 2011–2017 and recreational fishing in the Forster region is considered to be less than the densely populated Port Stephens region which has several boat ramps and a large influx of fishing tourists throughout the year.

Given that the *C*. *auratus* decline in Seal Rocks was predominantly legal sized fish (> 30 cm) and that compliance seizures by compliance interceptions were all of fish over 30 cm, it is unlikely that illegal fishers are taking under sized fish from this no-take area. Additionally, of the vessel interceptions, none were over the bag limit of 10 *C*. *auratus* per person. It is considered unlikely that the illegal fishers would keep undersize fish or exceed the bag limit of ten *C*. *auratus* as they run the risk of being caught by compliance staff traversing between the site and boat ramp or being investigated on return to the boat ramp. Whilst it is possible for fishers to provide a mistruth about the location where they caught the fish, it is much more difficult to avoid an infringement for possessing undersize fish or exceeding the bag limit as undersize fish and over ten fish is difficult to refute. Additionally, as the Seal Rocks no-take area has the largest mean size of fish over 30 cm, and a large proportion of fish are over the minimum size limit, there is no real need for fishers to keep undersize fish as they are likely to catch fish exceeding the minimum size when illegally fishing within the Seal Rocks no-take area. The decline in *C*. *auratus* abundance by 55% from 2011 to 2017 in the Seal Rocks no-take area strongly indicates that the amount of illegal fishing activity observed is reducing the potential effect size of this no-take reserve for *C*. *auratus*, hence, reducing the likelihood of it meeting its marine park objectives.

In NSW, the objectives of no-take areas (also known as sanctuary zones in NSW) are to provide the greatest level of protection to support conservation of biological diversity, maintenance of ecosystem integrity and function, enable scientific research and education, and provide for public appreciation, enjoyment, and cultural use (NSW DPI, 2017). As *C*. *auratus* are a dominant species on shallow reefs throughout eastern Australia, and important predators in the structure and function of shallow reef communities [32], the harvest of fish in no-take areas is likely to result in a reduction in the ecological benefits that would otherwise accrue. MPAs that fail to meet conservation or sustainability goals are often under resourced, particularly in regards to enforcement; contain insufficient no-take areas; and often have poor governance and minimal community involvement [52].

This study indicates there is a strong correlation between illegal recreational fishing within the Seal Rocks no-take area and a decline in a targeted fishery species. The abundance and size of *C*. *auratus* were found to decline within the Seal Rocks no-take area, whilst no decline was evident at surrounding fished locations or other no-take areas. The illegal fishing activity has led to the abundance of *C*. *auratus* within the Seal Rocks no-take area to decline to abundance levels that are now similar to that currently of nearby fished areas. The ongoing illegal fishing by recreational fishers in the Seal Rocks no-take area was demonstrated through the novel use of a long range surveillance camera. Hence, such illegal fishing is compromising the capacity of this no-take area in meeting its ecological, scientific and social objectives. In particular, illegal fishing results in difficulties in the interpretation of data collected from this scientific reference location. Increased education, improved use of camera technology, and increased resources to allow targeted compliance are essential to reduce the ongoing illegal fishing activity within MPAs in order to ensure that they achieve their stated objectives.
